# MicroRNA-941 Expression in Polymorphonuclear Granulocytes Is Not Related to Granulomatosis with Polyangiitis

**DOI:** 10.1371/journal.pone.0164985

**Published:** 2016-10-18

**Authors:** Jesper Brink Svendsen, Bo Baslund, Elisabeth Præstekjær Cramer, Nicolas Rapin, Niels Borregaard, Jack Bernard Cowland

**Affiliations:** 1 The Granulocyte Research Laboratory, Department of Hematology, National University Hospital, Copenhagen, Denmark; 2 Department of Rheumatology, National University Hospital, Copenhagen, Denmark; 3 The Finsen Laboratory, National University Hospital, Copenhagen, Denmark; Uniwersytet Gdanski, POLAND

## Abstract

Jumonji Domain-Containing Protein 3 (JMJD3)/lysine demethylase 6B (KDM6B) is an epigenetic modulator that removes repressive histone marks on genes. Expression of KDM6B mRNA is elevated in leukocytes from patients with ANCA-associated vasculitis (AAV) and has been suggested to be the reason for higher proteinase 3 (PR3) mRNA expression in these cells due to derepression of *PRTN3* gene transcription. MicroRNA-941 (miR-941) has been shown to target KDM6B mRNA and inhibit JMJD3 production. We therefore investigated whether polymorphonuclear granulocytes (PMNs) from patients suffering from granulomatosis with polyangiitis (GPA) have lower expression of miR-941 than healthy control donors as a biological cause for higher JMJD3 levels. We found no significant difference in the degree of maturation of PMNs from GPA patients (n = 8) and healthy controls (n = 11) as determined from cell surface expression of the neutrophil maturation marker CD16 and gene expression profile of *FCGR3B*. The expression of *PRTN3* and *KDM6B* mRNAs and miR-941 was not significantly different in GPA patients and healthy controls. Transfection of pre-miR-941 into the neutrophil promyelocyte cell line PLB-985 cells did not result in reduction of the KDM6B mRNA level as shown previously in a hepatocellular carcinoma cell line. The amount of PR3 in PMNs from GPA patients and healthy controls was comparable. In conclusion, we found that PRTN3 mRNA, KDM6B mRNA, and miR-941 expression levels in PMNs do not differ between GPA patients and healthy controls, and that miR-941 does not uniformly regulate KDM6B mRNA levels by inducing degradation of the transcript. Thus, decreased miR-941 expression in PMNs cannot be part of the pathogenesis of GPA.

## Introduction

Granulomatosis with polyangiitis (GPA), formerly known as Wegener’s granulomatosis, is an anti-neutrophil cytoplasmic antibody (ANCA)-associated vasculitis (AAV). GPA is a granulomatous inflammation involving the respiratory tract and a necrotizing vasculitis that affects small- to medium-sized blood vessels. Necrotizing glomerulonephritis is common in GPA [1]. The majority of GPA patients are proteinase 3 (PR3)-ANCA positive, a small group are myeloperoxidase (MPO)-ANCA positive, while only few are ANCA-negative [2]. There are ongoing investigations on the causal relation to aberrant expression of PR3 versus MPO, and consensus seems to be that membrane-bound PR3 (mPR3) is a significant risk factor for the development of PR3-ANCA disease, e.g. GPA, and even more significant in relapse [3,4].

MicroRNAs (miRNAs) are small (~22 nt), non-coding RNAs that play a major role in many cellular processes such as differentiation and proliferation [5,6]. miRNAs bind to the 3’-untranslated regions (3’-UTRs) of their target-mRNAs with decreased translation and/or destabilization and degradation of the targeted mRNAs as consequence [5–7]. In a previous study of the miRNA expression profile during granulopoiesis we found 135 differentially regulated miRNAs by microarray analysis including miR-941 [8].

It has been shown that there is a higher expression of the transcripts for PR3 and the epigenetic regulator JMJD3 in total leukocytes from AAV patients compared to healthy controls and it was hypothesized that removal of the inhibitory epigenetic mark H3K27me_3_ on the *PRTN3* gene by JMJD3 was the reason for higher PRTN3 mRNA expression [9]. Concomitantly, it has been demonstrated that expression of miR-941 and JMJD3 mRNA was lower and higher, respectively, in hepatocellular carcinoma (HCC) tissue compared to adjacent healthy tissue, indicating that miR-941 targets JMJD3 mRNA. In accordance, miR-941 was shown to cause degradation of JMJD3 mRNA in an HCC cell line [10]. Based on these findings, we decided to examine whether low levels of expression of miR-941 in PMNs from GPA patients could be the reason for higher JMJD3 mRNA levels reported previously in total leukocytes from AAV patients [9].

## Materials and Methods

### Blood and bone marrow samples

Bone marrow aspirates and peripheral blood samples from patients and healthy controls (HC) were obtained after informed and written consent according to permissions H-1-2011-65 and H-2-2009-103 in compliance with the Helsinki Declaration and guidelines from the local ethics committee of the Capital Region of Denmark.

### Patient inclusion

GPA patients (n = 8) referred to the Department of Rheumatology, Rigshospitalet, University of Copenhagen, were included in the study based on clinical presentation and recognizable active disease confirmed by Birmingham Vasculitis Activity Score (BVAS). Healthy control donors (n = 11) were staff members.

### Isolation of total leukocytes, PMNs, and monocytes from peripheral blood and neutrophil precursors from bone marrow

Granulocytes were isolated from peripheral blood as described in [11]. Briefly, erythrocytes were sedimented by 2% Dextran and the supernatant containing “total leukocytes” separated on a density gradient by centrifugation in Lymphoprep^™^ (Axis-Shield). Monocytes were purified from the top layer by immunomagnetic cell sorting (MACS^™^ (Miltenyi)), using murine anti-CD14 antibodies (eBioscience 14-0149-82) and bead-labeled rat-anti-mouse antibodies (MACS 130-047-101). Residual erythrocytes in the granulocyte cell pellet (after density centrifugation) were destroyed by hypotonic lysis. Eosinophils were removed by MACS using anti-CD49d antibodies (14-0499-82, eBioscience) and bead-labeled rat-anti-mouse antibodies (MACS 130-047-101). Purity of the isolated neutrophils and their stage of maturation was evaluated by inspection of May-Grünwald-Giemsa (MGG) stained cytospins and expression of maturation markers by flow cytometric analysis and quantitative real-time PCR (qRT-PCR).

Expression of PRTN3 mRNA, KDM6B mRNA, and miR-941 was examined in neutrophil precursors from human bone marrow aspirates isolated by density centrifugation on a Percoll gradient followed by immunomagnetic depletion of non-neutrophil cells as described in [11]. The three bone-marrow-derived cell populations isolated in this manner consisted of myeloblasts & promyelocytes (MB&PM), myelocytes & metamyelocytes (MC&MM), and band cells & segmented cells (BC&SC), respectively. Total RNA from these cells was used for mRNA and miRNA array analysis [8,12].

### Flow cytometry analysis

Flow cytometry was carried out on a FACS-Calibur (BD Biosciences) followed by data analysis on Cell Quest (BD Biosciences) and FlowJo (FlowJo, LLC) software. Cells were stained with a FITC-labeled antibody against CD16 (555407) using mouse-anti-human IgG1к (555749) as isotype control (both BD Biosciences).

### Cell culture and transfections

PLB-985 cells (DSMZ ACC-139) were grown in RPMI 1640 (Gibco BRL) supplemented with 10% heat-inactivated fetal bovine serum (FBS) (Gibco BRL) and 1% 100 U/ml penicillin and 100 μg/ml streptomycin (P/S) (Gibco BRL). Electroporation of 2 x 10^6^ PLB-985 cells was performed according to the manufacturers recommendations (AMAXA) using program T-019 with 30 pmole pre-miR-941 (PM 12273), pre-miR negative control #1 (AM17110), siRNA against KDM6B (Silencer Select P/N 4392420), or siRNA negative control #1 (Silencer Select P/N 4390843), respectively—all from Applied Biosystems. Transfections were done in triplicate. Cells were analyzed 24 hours post-transfection.

### Quantitative real-time polymerase chain reaction (qRT-PCR)

RNA was extracted using TRIzol^®^ Reagent (Invitrogen) according to the manufacturer’s instructions. qRT-PCR was performed on an MX 3000P real-time PCR system (Stratagene) as described earlier [8] with TaqMan mRNA assays for PRTN3 (Hs01597752_m1), KDM6B (Hs00996325_g1), and FCGR3B (Hs04334165_m1) using β-actin (4326315E) for normalization and the miRNA assay for miR-941 (002183) using RNU6B (001093) for normalization—all from Applied Biosystems.

### Western blot

Western blotting was performed as described in [13]. Purified neutrophils from GPA patients and HCs were resuspended in Laemmli buffer and 2 x10^5^ cells analyzed on a 4–12% NuPage Bis-Tris gradient gel (Invitrogen). Antibodies: Rabbit anti-human proteinase 3 (1:250; Abcam 103632) succeeded by HRP-conjugated goat-anti-rabbit (1:1000; DAKO P0448). The membrane was stripped and reprobed with goat-anti-human-β-actin (1:500; Santa Cruz sc-1616) succeeded by HRP-conjugated rabbit-anti-goat (1:1000; DAKO P0449). The membrane was developed by chemiluminiscence using SuperSignal West Pico (Pierce) and analyzed on a Bio-Rad Chemidoc (Bio-Rad). Quantification of band intensities was performed with ImageLab software (Bio-Rad).

### Gene expression data analysis

Gene expression profiles for PR-3-ANCA samples [9] (dataset 1) (kindly provided by Dr. Ciavatta) were analyzed together with samples from normal hematopoiesis taken from [14], GEO accession number GSE42519 (dataset 2). Shortly, after normalization at the probe sets level with RMA [15], both dataset were merged and gene expression data transformed to ranks. A principal component analysis was then performed on the merged dataset to locate the position of the ANCA samples within the normal hematopoietic hierarchy. Genes used to construct the PCA were selected using limma [16] by performing all pairwise comparisons between all normal population in the PCA space (P < 0.05, top twenty probe sets for each comparison).

### Statistical analysis

Mean fluorescence intensity (MFI) data were analyzed by Mann-Whitney U-test. For qRT-PCR data, unpaired Student´s t-test was applied on ΔCt values as they meet the requirements for parametric analysis. The relative expression is shown in graphs together with medians and interquartile ranges. PRTN3 intensity on Western blot is analyzed by Mann-Whitney U-test. Significance level was set to 0.05, and statistical analysis was performed using GraphPad Prism 5.0 (GraphPad Software).

## Results

### Expression profiles of miR-941, PRTN3 mRNA, and KDM6B mRNA in normal granulopoiesis

**Fig 1:**
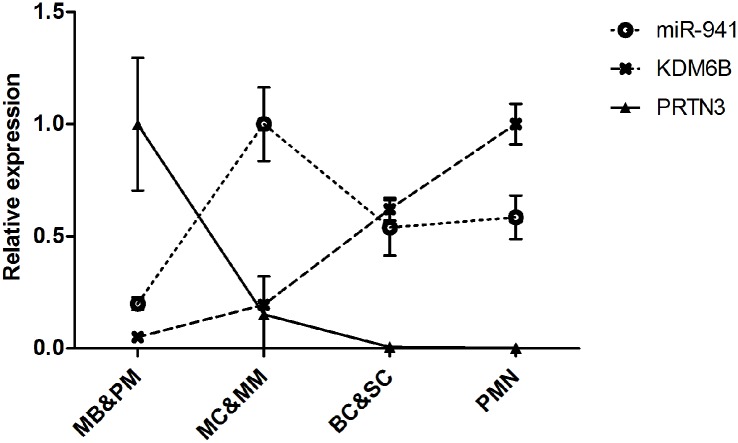
Expression of miR-941, KDM6B mRNA, and PRTN3 mRNA during human granulopoiesis. Relative expression of miR-941 (circles), KDM6B mRNA (crosses), and PRTN3 mRNA (triangles) in myeloblasts and promyelocytes (MB&PM), myelocytes and metamyelocytes (MC&MM), band cells and segmented cells (BC&SC), and PMNs, respectively. Data are from our previous global expression analyses of mRNAs [12,17] and miRNAs [8] during granulopoiesis. For all three RNAs, data are shown relative to the cell population with the highest expression, which is given the value 1. Error bars are mean ± SD.

In a previous study, we examined the miRNA expression profile during normal granulopoiesis and found by miRNA microarray analysis that miR-941 was expressed at low levels in the most immature precursors myeloblasts/promyelocytes (MB&PM), reaching a high expression level in the myelocytes/metamyelocytes (MC&MM), i.e. at the stage where cell proliferation ceases and terminal differentiation commences, followed by an intermediate expression level in band cells/segmented cells (BC&SC) and PMNs [8] (Fig 1). This expression pattern was validated by qRT-PCR (data not shown).

Using the online miRNA-target prediction software TargetScan (www.targetscan.org), the epigenetic regulator JMJD3/KDM6B is predicted as a miR-941 target. In a recent publication, it was shown that the mean expression level of KDM6B mRNA is almost two-fold higher in total leukocytes from AAV patients compared to healthy controls, whilst there is an almost twentyfold increase in mean PRTN3 mRNA expression level in total leukocytes from AAV patients compared to healthy controls (HCs) [9]. The authors concluded that a rise in JMJD3 expression causes a selective derepression of the *PRTN3* promoter through demethylation of the repressive epigenetic mark histone 3 lysine 27 (H3K27), thereby causing higher PR3 expression in mature neutrophils of AAV than in healthy individuals. Consequently, more PR3 may be expressed on the PMN membrane and trigger development of AAV [9].

The expression profiles of PRTN3 and KDM6B in healthy individuals were retrieved from the HemaExplorer database [17]. KDM6B expression is shown to gradually increase during granulopoiesis, reaching the highest level of expression in the mature PMN whereas the transcript level for PRTN3 peaks in the most immature precursors (MB&PM) and thereafter diminish during granulopoiesis, reaching an almost unmeasurable level in band cells (BC) and onwards (Fig 1).

### PMNs from GPA patients and healthy controls are of equal maturity

**Fig 2:**
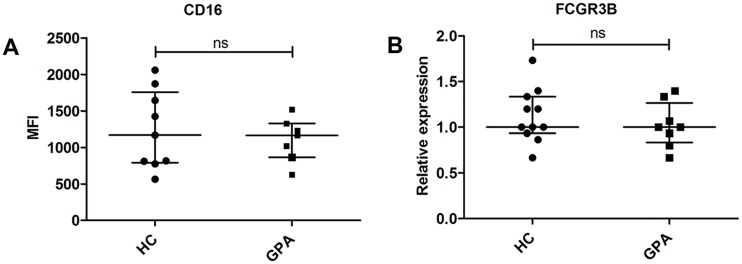
Expression of PMN maturation markers. **(A)** Mean fluorescence intensities (MFI) for the cell surface marker CD16 and **(B)** FCGR3B mRNA expression levels in PMNs from healthy controls (HC, circles) and patients with granulomatosis with polyangiitis (GPA, squares). Expression of FCGR3B is shown relative to the median value for the HC group. Horizontal lines indicate medians, and error bars depict interquartile ranges.

Due to the variability in expression levels of miR-941, KDM6B mRNA, and PRTN3 mRNA during granulopoiesis, we decided to evaluate the stage of maturation of neutrophils from GPA patients compared to healthy controls to ensure that the PMNs used for comparison of RNA expression levels were of equal maturity (Table 1). This was evaluated by measuring the expression level of the well characterized cell surface maturation marker CD16, which increases with maturation (Fig 2A) [18]. We also measured the mRNA expression profile of the FCGR3B transcript (Fig 2B) which has a very prominent maturation-dependent expression pattern (S1 Fig). There were no significant differences in expression of these maturation markers in PMNs from GPA patients and healthy controls (Fig 2). The morphology of the cells on MGG-stained cytospins of all PMN preparations supported this conclusion (data not shown) and allowed us to compare the gene expression levels from the PMNs.

**Table 1:** Data table of patients and healthy controls.

**Patient**	**1**	**2**	**3**	**4**	**5**	**6**	**7**	**8**			
Age, years	57	48	64	40	90	60	24	43			
Gender:(male (M) / female (F))	M	M	M	F	F	F	F	M			
BVAS score/years of disease (0 = new patient)	19/0	18/0	6/24	20/0	20/0	15/0	9/0	10/7			
Steroid treatment before sample collection (days)	15	2	1	0	>30	>30	3	3			
Steroid daily dosis (mg)	50	75	75	0	10	75	25	50			
Other immunotherapy, drug^a^	C9	0	0	0	0	0	MT2	MT0			
WBC count, x10^9^ cells/liter (normal 4.5–13.5)	10.7	16.6	15	7.9	15.1	17.5	9.7	16.3			
Neutrophil count, x10^9^ cells/liter (normal 2.0–8.0)	8.7	14.6	12.2	6.9	13.3	11.2	7.1	13.8			
**Healthy control**	**1**	**2**	**3**	**4**	**5**	**6**	**7**	**8**	**9**	**10**	**11**
Age, years	27	55	56	32	33	22	31	38	26	25	31
Gender:(male (M) / female (F))	M	M	F	F	F	F	M	F	M	F	F

^a^C9 = Cyclophosphamide nine days prior, MT2 = Methotrexate 2 days in advance, MT0 = Methotrexate immediately before sample collection

### Expression of PRTN3 mRNA and KDM6B mRNA is the same in PMNs from GPA patients and healthy controls

**Fig 3:**
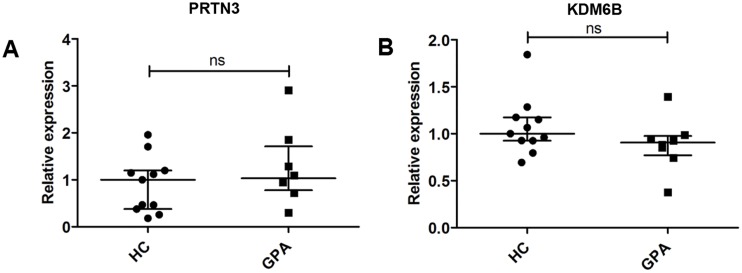
Expression of PRTN3 and KDM6B mRNAs in PMNs. **(A)** Relative expression of PRTN3 mRNA and **(B)** KDM6B mRNA in PMNs from healthy controls (HC, circles) and patients with granulomatosis with polyangiitis (GPA, squares). Expression is shown relative to the median value for the HC group. Horizontal lines indicate medians, and error bars depict interquartile ranges.

We next examined whether we could reproduce the differences in PRTN3 and KDM6B mRNA expression reported in total leukocytes from AAV patients and HCs [9] by examining the levels of these mRNAs in our purified PMNs from GPA patients and HCs. Contrary to the earlier study [9], we found no significant difference in the expression of PRTN3 mRNA in PMNs purified from HCs and GPA patients (Fig 3A). This observation does not support a higher expression of the *PRTN3* gene in PMNs of GPA patients as previously shown in leukocytes of AAV patients. Next, we examined the expression of KDM6B mRNA in GPA patients and HCs to determine whether we could reproduce earlier findings of higher levels of JMJD3 in AAV patients [9]. Also in this case, no significant difference in the expression of KDM6B mRNA in the PMN fraction of HCs versus the PMN fraction of GPA patients was observed (Fig 3B).

### miR-941 does not induce degradation of KDM6B mRNA in PLB-985 cells

**Fig 4:**
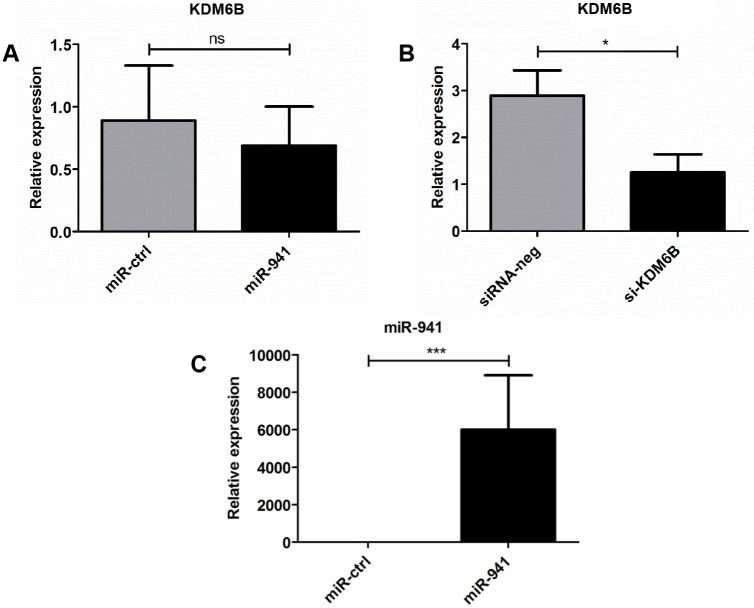
Effect of miR-941 on KDM6B mRNA stability in PLB-985 cells. **(A and B)** Relative expression of KDM6B mRNA in PLB-985 cells transfected with (A) a negative control miRNA (miR-ctrl) and miR-941 (miR-941) showing no significant difference and (B) with negative si-RNA (siRNA-neg) and a siRNA against KDM6B (si-KDM6B) displaying significant knock-down of KDM6B mRNA. **(C)** Relative expression of miR-941 in the PLB-985 transfection shown in (A). All transfections were done in triplicate. Horizontal lines indicate medians, and error bars depict interquartile ranges (* p < 0.05, *** p < 0.001).

As miR-941 is a potential regulator of JMJD3 expression in PMNs we examined whether transfection of PLB-985 cells would cause degradation of the KDM6B mRNA as in HCC [10]. This was not the case as comparable amounts of KDM6B mRNA was measured in both pre-miR-941 and control-miRNA-transfected cells (Fig 4A). As control, we also transfected the cells with another small RNA, a siRNA against KDM6B mRNA, and could in this case demonstrate a more than 50% knockdown of KDM6B mRNA (Fig 4B). To validate that the PLB-985 cells were indeed transfected with pre-miRNA we quantified the level of miR-941 in the cells by qRT-PCR and found a ~5.000 fold higher level of miR-941 expression in pre-miR-941-transfected cells compared to control-transfected cells (Fig 4C). The same results were obtained following transient transfection of human embryonic kidney cells (HEK-293) with miR-941 and siRNA against KDM6B (data not shown).

### Expression of miR-941 and proteinase 3 is the same in PMNs from GPA patients and healthy controls

**Fig 5:**
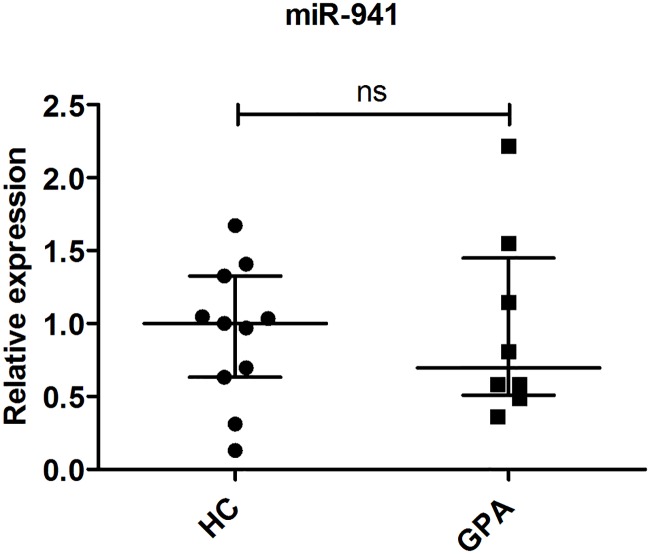
miR-941 expression in PMNs. Relative expression of miR-941 in PMNs from healthy controls (HC, circles) and patients with granulomatosis with polyangiitis (GPA, squares). Expression of miR-941 is shown relative to the median value for the HC group. Horizontal lines indicate medians, and error bars depict interquartile ranges.

**Fig 6:**
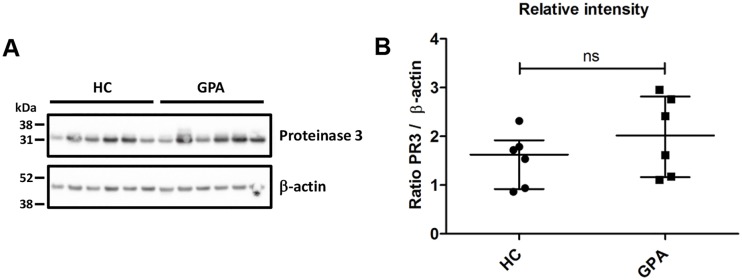
Expression of proteinase 3 in PMNs. **(A)** Western blot of total PMN protein from 6 healthy controls (HC) and 6 patients with granulomatosis with polyangiitis (GPA). **(B)** Relative intensities of proteinase 3 following normalization to the corresponding β-actin intensity for the 6 HCs and 6 GPA patients. Horizontal lines indicate medians, and error bars depict interquartile ranges.

Although we demonstrated that miR-941 does not cause degradation of KDM6B mRNA in PLB-985 cells, it is still possible that it could affect translation of KDM6B mRNA if miR-941 were differentially expressed in PMNs from GPA patients and HCs. This, however, was also not the case as miR-941 expression in our two groups showed no significant difference (Fig 5). Quantification of proteinase 3 in PMNs from six GPA patients and six healthy controls did not show any significant difference in protein levels (Fig 6).

**Fig 7:**
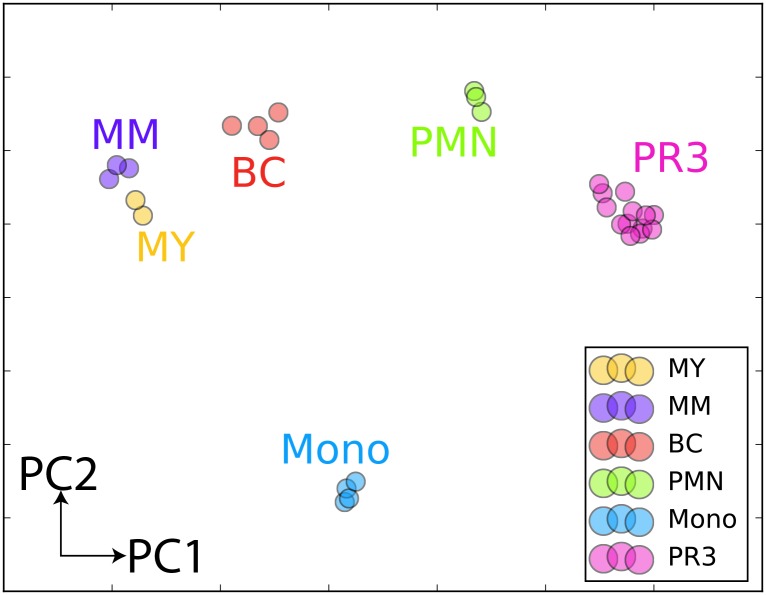
Principal component analysis of total leukocytes from PR3-ANCA patients. Gene expression profiles for PR3-ANCA samples [9] were analyzed together with samples of normal neutrophil precursors and monocytes taken from [14]. Cell populations represented are: MY: myelocytes, MM: metamyelocytes, BC: band cells, PMN: polymorphonuclear granulocytes, Mono: monocytes, PR3: PR3-positive GPA leukocytes.

One possible cause for the discrepancies between the observations in our study and the previously published data [9] is that in the latter case expression of PRTN3 and KDM6B mRNAs was measured in a total leukocyte population rather than in highly purified neutrophils. To this end, we decided to perform a bioinformatics analysis of the array expression data of PR3-ANCA positive patients from the Ciavatta study [9] and compare this to array expression data from purified neutrophil precursor populations as well as monocytes from peripheral blood [14]. A principal component analysis (PCA) demonstrated an expression profile of the PR3-ANCA patients different from that of PMNs and monocytes (Fig 7). We have found, with differential expression analysis, genes that are clearly characteristic for each step of differentiation. Using the resulting genes list and plotting their ranks across several hematopoietic populations clearly shows that GPA-PR3 samples are not similar to either PMNs or monocytes from peripheral blood (S2 Fig). The relative expression of PRTN3 mRNA in total leukocytes, monocytes, and PMNs was determined by quantitative real-time PCR and demonstrated a generally higher expression in total leukocytes than in neutrophils and an almost absence of PRTN3 mRNA in monocytes (S3 Fig).

Together, our data do not support a model where higher expression of PRTN3 and KDM6B mRNA in PMNs from GPA patients is a cause for disease development, nor that miR-941 is a regulator of KDM6B mRNA stability in all cellular settings.

## Discussion

In this study we wished to examine whether a lower level of miR-941 expression could be the cause of the higher level of KDM6B mRNA expression previously reported in leukocytes from AAV patients and offered as explanation for the elevated PRTN3 mRNA levels measured in these cells [9]. As neutrophil granulocytes are, by far, the main source of PR3, we decided to conduct our analysis on purified PMNs rather than total leukocytes. In this setting, we could not reproduce the differences in KDM6B mRNA and PRTN3 mRNA expression between GPA patients and healthy donors, previously reported for total leukocytes in AAV patients [9]. As JMJD3, on the other hand, probably is expressed in all leukocyte subpopulations, differences in the cellular composition of total leukocytes between healthy donors and GPA patients due to differences in sample preparation or as a result of the disease could explain the slightly increased KDM6B mRNA levels previously measured for the GPA patients. The importance of removing non-neutrophils is illustrated in an earlier work where we found that C/EPB-ε, in contrast to previous reports, was not expressed in PMNs, but rather in the small amount of contaminating eosinophils [11]. Regarding the almost 20-fold higher expression of PRTN3 mRNA in AAV patients reported by Ciavatta et al. [9], this could be due to the presence of immature neutrophil precursors in the total leukocyte population. The expression of PRTN3 mRNA peaks in MB&PMs in accordance with synthesis and storage of PR3 in the azurophil granules of the neutrophil granulocyte (Fig 1) [19]. The expression of PR3 then tapers off to an almost undetectable level in mature neutrophils. A minor contamination of the AAV samples with slightly less mature neutrophil precursors may thus give the impression of higher PR3 mRNA expression in PMNs. Our PCA analysis demonstrated that the gene expression profile of the PR3-ANCA samples from the Ciavatta study [9] differed from that of PMNs (Fig 7). Expression of PRTN3 was generally higher in the total leukocyte fraction than in purified neutrophils (S3 Fig) and almost absent in monocytes (Fig 7 and S3 Fig). The presence of a few immature neutrophils in the total leukocyte fraction could thus be a possible explanation for the slightly elevated PRTN3 expression in PR3-ANCA leukocytes. This potential source of error was eliminated in our study by examining only highly purified PMNs. Another factor that could explain the above discrepancies is that the data reported by Ciavatta et al. [9] included both PR3- and MPO-ANCA patients whereas we have focused solely on PR3-positive (GPA) patients.

We found that miR-941 does not degrade KDM6B mRNA in PLB-985 cells, contrary to the findings in HCC cells [10]. This demonstrates that KDM6B mRNA degradation is not a universal mechanism for miR-941. We do not know whether miR-941 can degrade KDM6B mRNA in PMNs or whether it simply represses translation, but since we did not find any differences in expression neither of PRTN3 mRNA, KDM6B mRNA, nor miR-941, this indicates that miR-941 does not play a role in GPA pathogenesis.

As mentioned above, PR3 is expressed in MBs and PMs and stored in azurophil granules [19]. A role for JMJD3 as epigenetic regulator of PRTN3 gene expression could therefore be envisioned at the PM/MC transition where overexpression of JMJD3 could delay an epigenetic silencing of the *PTNR3* gene with erroneous expression of PRTN3 mRNA and PR3 protein in MCs where the specific granules are formed. An examination of the effect of JMJD3 on *PRTN3* gene expression in immature neutrophil precursors might therefore be of potential interest as a model for GPA disease development.

## Supporting Information

S1 Fig Expression profile of FCGR3B mRNA during normal granulopoiesis. Expression of FCGR3B mRNA in early promylocytes (early_PM), late promyelocytes (late_PM), myelocytes (MY), metamyelocytes (MM), band cells (BC), PMNs, and monocytes (Mono). Expression data are from the HemaExplorer database [17] demonstrating a significant upregulation of FCGR3B mRNA in mature PMNs.
(TIF)

S2 Fig Expression profiles for genes in hematopoietic populations. Genes found to be differentially expressed in specific populations of cells and expression levels in these. Cell populations represented are: MY: myelocytes, MM: metamyelocytes, BC: band cells, PMN: polymorphonuclear granulocytes, Mono: monocytes, PR3: PR3-positive GPA leukocytes.
(TIF)

S3 Fig Expression of PRTN3 mRNA in total leukocytes, monocytes, and PMNs. Relative expression of PRTN3 mRNA in total leukocytes (TL), monocytes (MC), and PMNs (PMN) from peripheral blood of **(A)** five healthy controls (HC) and **(B)** two patients with granulomatosis with polyangiitis (GPA). PRTN3 expression is shown relative to the expression in TL, which is given the value 1. Error bars are mean ± SD.
(TIF)
