# Supplementary figures and images for: MicroRNA-941 Expression in Polymorphonuclear Granulocytes Is Not Related to Granulomatosis with Polyangiitis
Source: PLoS One. 2016 Oct 18;11(10):e0164985. doi: 10.1371/journal.pone.0164985 (PMC5068789; doi:10.1371/journal.pone.0164985)

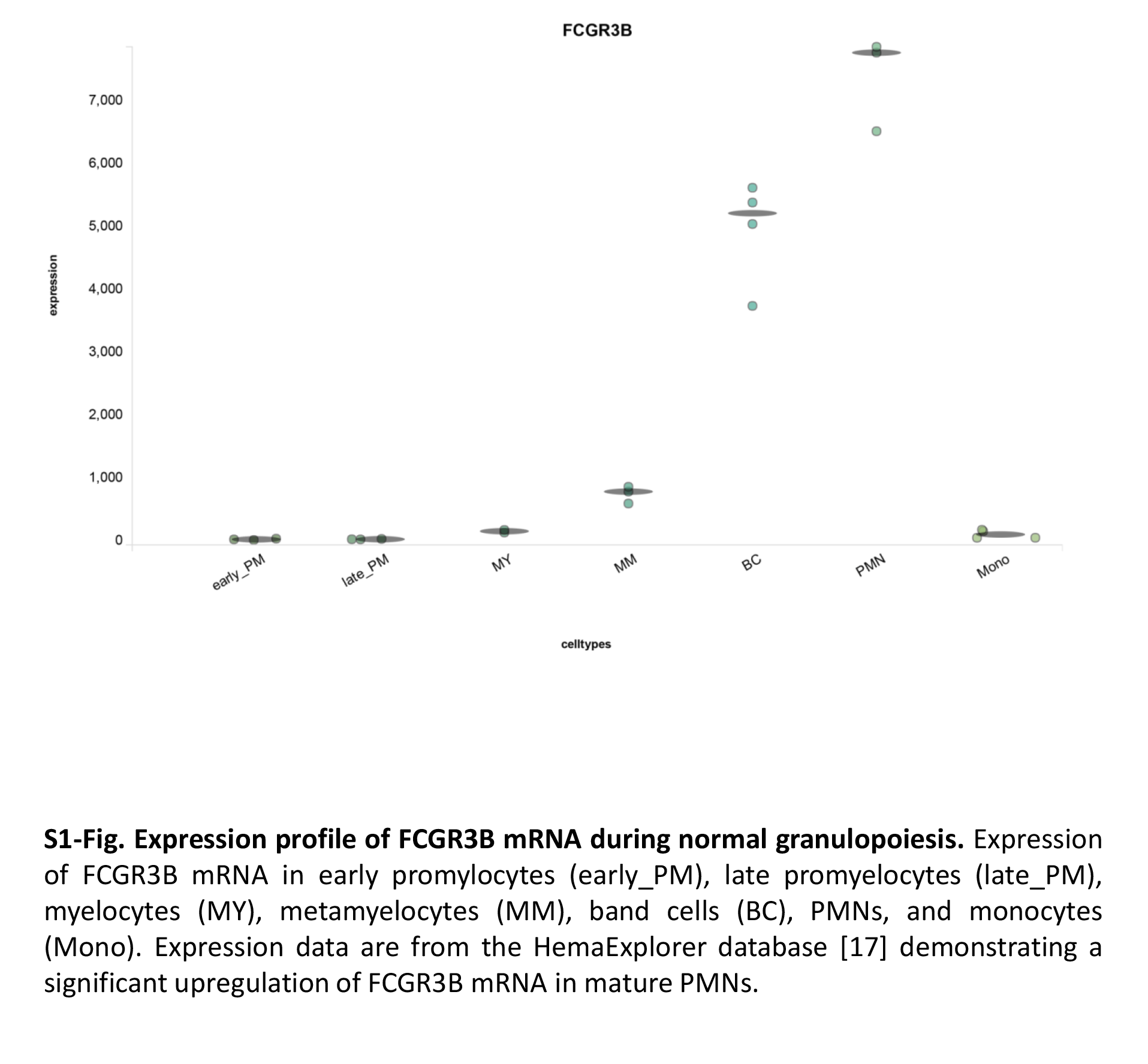

Supplement: S1 Fig — Expression of FCGR3B mRNA in early promylocytes (early_PM), late promyelocytes (late_PM), myelocytes (MY), metamyelocytes (MM), band cells (BC), PMNs, and monocytes (Mono). Expression data are from the HemaExplorer database [17] demonstrating a significant upregulation of FCGR3B mRNA in mature PMNs. (TIF) [file pone.0164985.s001.tif]

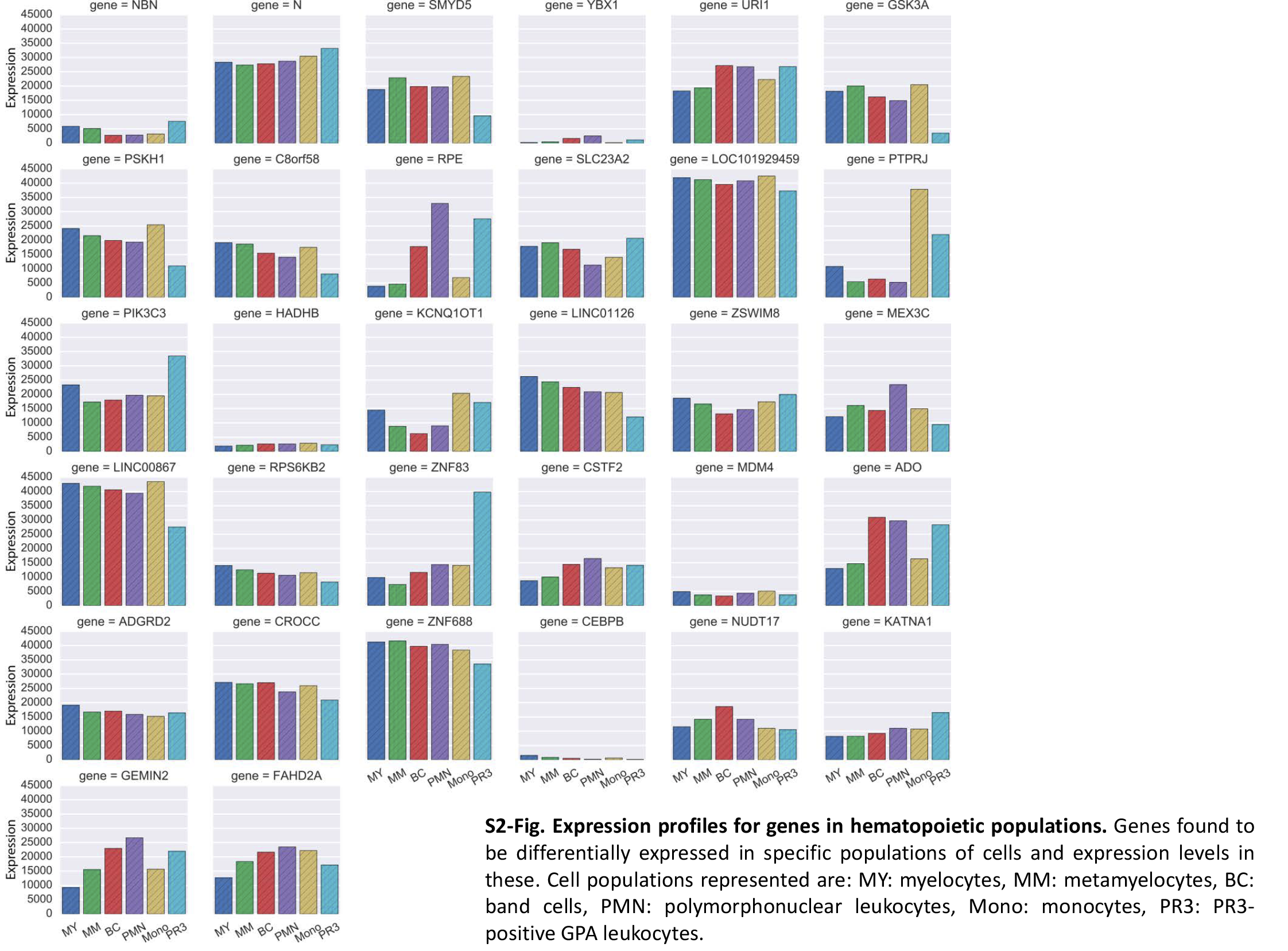

Supplement: S2 Fig — Genes found to be differentially expressed in specific populations of cells and expression levels in these. Cell populations represented are: MY: myelocytes, MM: metamyelocytes, BC: band cells, PMN: polymorphonuclear granulocytes, Mono: monocytes, PR3: PR3-positive GPA leukocytes. (TIF) [file pone.0164985.s002.tif]

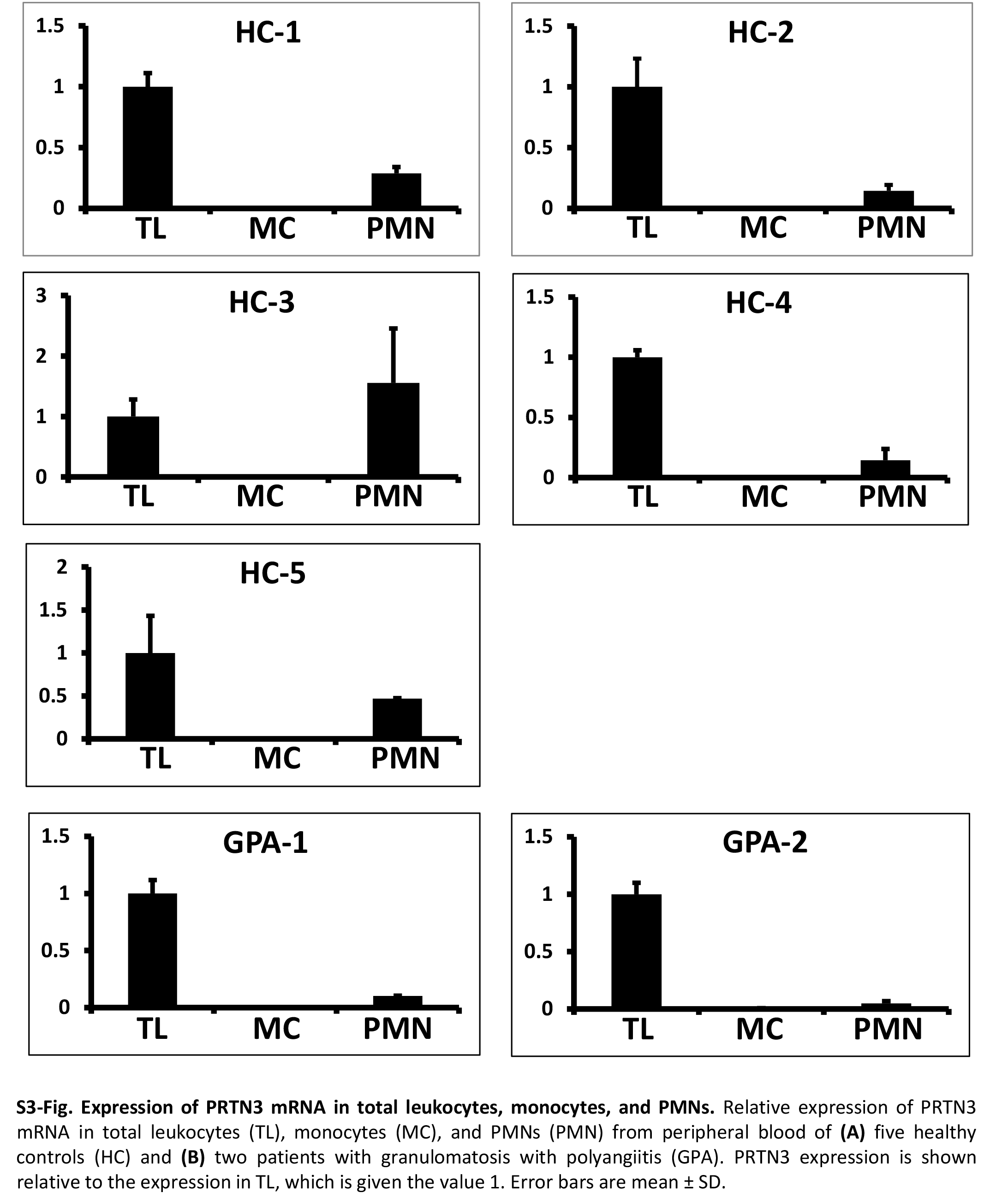

Supplement: S3 Fig — Relative expression of PRTN3 mRNA in total leukocytes (TL), monocytes (MC), and PMNs (PMN) from peripheral blood of (A) five healthy controls (HC) and (B) two patients with granulomatosis with polyangiitis (GPA). PRTN3 expression is shown relative to the expression in TL, which is given the value 1. Error bars are mean ± SD. (TIF) [file pone.0164985.s003.tif]
